# Right sided Bochdalek diaphragmatic hernia appeared as a life-threatening event in an infant: a case report

**DOI:** 10.11604/pamj.2021.38.150.28044

**Published:** 2021-02-10

**Authors:** Charikleia Demiri, Vasilios Mouravas, Vasilios Lambropoulos, Chrysostomos Kepertis, Kleanthis Anastasiadis, Ioannis Spyridakis

**Affiliations:** 1 Department of Paediatric Surgery, Medical School, Aristotle University of Thessaloniki, General Hospital of “Papageorgiou”, Thessaloniki, Greece

**Keywords:** Right sided Bochdalek hernia, life-threatening condition, paediatric surgery, case report

## Abstract

We report a case of a 5-month-old female infant who presented with a cardiorespiratory distress and shock. After thoracic computed tomography (CT) scan, a right sided Bochdalek hernia was diagnosed with massive herniation of the abdominal viscera causing mediastinal shift. The girl underwent emergency laparotomy, which confirmed the right sided diaphragmatic hernia with herniation of small bowel and colon. After reduction of herniated contents, the defect in the diaphragm was closed. The patient had an uneventful post-operative cause. This case demonstrates that an undiagnosed Bochdalek hernia can appear with such a severe, life-threatening and misleading presentation.

## Introduction

Bochdalek diaphragmatic hernia (BDH), which was first described in 1848 by the Czech anatomist and pathologist, Vincenz Alexander Bochdalek, is an anatomical defect at the posterolateral part of the diaphragm [1]. Congenital diaphragmatic hernia (CDH) occurs in less than 1 to 5 in 10,000 births, typically presents in the first few hours of life with respiratory distress due to pulmonary hypoplasia and is rarely late-presenting with an estimated incidence of 2.6-11% [2]. Late presented CDH is characterized by nonspecific symptoms such as recurrent pulmonary infections or atypical gastrointestinal symptoms which vary from gastroesophageal reflux disease to severe and life-threatening conditions such as gastric volvulus, small bowel obstruction, incarceration, or strangulation [2,3]. The prenatal ultrasonography may not reveal the presence of this congenital anomaly and the literature indicates that the time or age at the diagnosis and the severity of clinical presentations cannot be predicted [4-6]. Herein, we describe a case of a 5-month-old female infant with an undiagnosed Bochdalek hernia (BH) who was admitted to our paediatric surgery emergency department by her parents, presenting disorientating signs and symptoms.

## Patient and observation

An otherwise healthy 5-month-old female infant, the first child of two young parents without medical problems in their past medical history or a history of congenital malformation in their families, was admitted to our paediatric emergency department. Her parents complained about remarkable anxiety and crying of the patient the last two hours. The mother described that the little patient encountered difficulty in the bowel movements the last two days and had an episode of vomiting the same day. Last but not least, the parents referred that the child had a head injury three hours ago as a result of falling from a sitting position backwards on a soft carpet. On the arrival, the oxygen saturation was 99%, the heart rate was 164 beats per minute and the temperature was 36 degrees of Celsius. The child was first examined by a paediatrician who asked a paediatric surgery consultation due to the referred head injury. From the paediatric surgery clinical examination and estimation, the Glasgow Coma Scale was 14, there were no palpable or visible lesions on the head and the eye´s pupils reacted to the light symmetrically. The abdominal auscultation revealed absence of bowel sounds, but no visible flatulence was present. From the abdominal palpation which was easily conducted, the abdomen was soft and there were not clinical signs of peritoneal irritation. The rectal digital examination had as a result an explosive evacuation of the bowel with diarrhea and was negative for blood.

**Figure 1:**
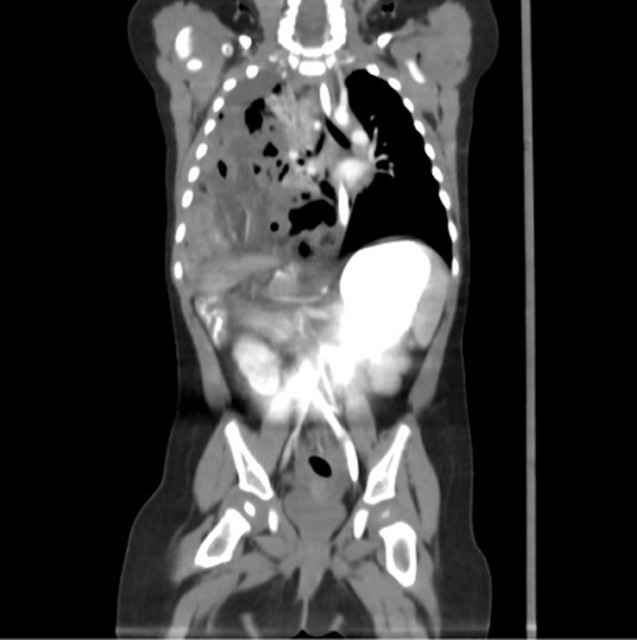
coronary axis CT: the right chest cavity almost completely occupied by loops of the gastrointestinal tract and intrathoracic projection of the mesenteric vessels

During the examination, the child presented intermittent peripheral acrocyanosis and because of the quick deterioration of the clinical condition, we immediately catheterized a peripheral vein, took blood samples and tried to make a chest and abdominal x-ray. The mother accidentally breastfed the child while waiting for the x-ray to be done and spontaneously the child vomited and lost consciousness. The patient was transferred to the shock room without a diagnosis establishment because the plain x-ray was not performed. An anesthesiologist was called, the child was intubated and after the vital signs’ stabilization and a nasogastric tube insertion, because of the very disorienting medical history and the wide spectrum of the differential diagnosis which included both brain injury, undiagnosed heart disease and gut volvulus, which means that we could not focus our diagnosis on which pathology gave those signs and symptoms, the baby underwent an immediate full body computed tomography scan (CT scan). From the first axial images the diagnosis was established (Figure 1). The right chest cavity was almost completely occupied by loops of the gastrointestinal tract, which presented local thickening of their wall and fluid elements that misaligned the right lung resulting in passive atelectasis of the biggest part of the right lower lobe. Simultaneously, the CT scan revealed intrathoracic projection of the mesenteric vessels and a large displacement of the vascular structures of the mediastinum. The liver was intact, there were not any other lesions of the abdominal viscera and the brain CT scan was normal. The diaphragmatic hernia diagnosis was established and the patient was immediately transferred to the operation theater and underwent an exploratory laparotomy after obtaining the parent´s informed consent.

**Figure 2:**
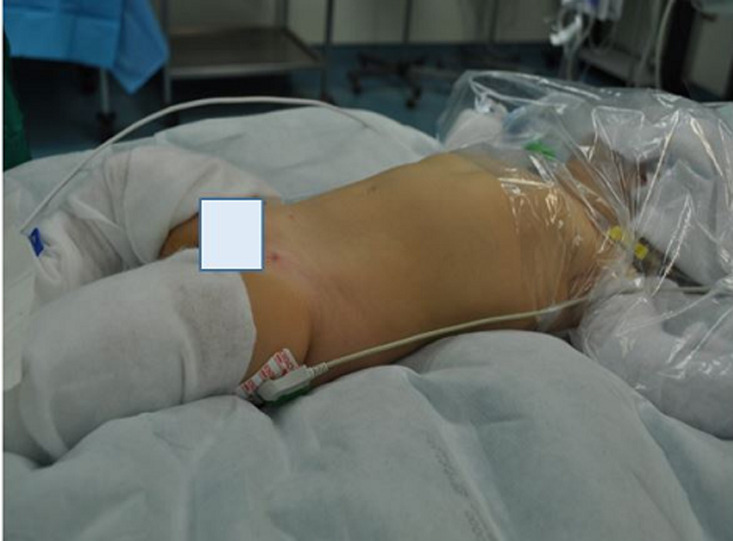
after the induction of anesthesia the scaphoid abdomen of the patient is obvious (patient's genitalia are covered)

**Figure 3:**
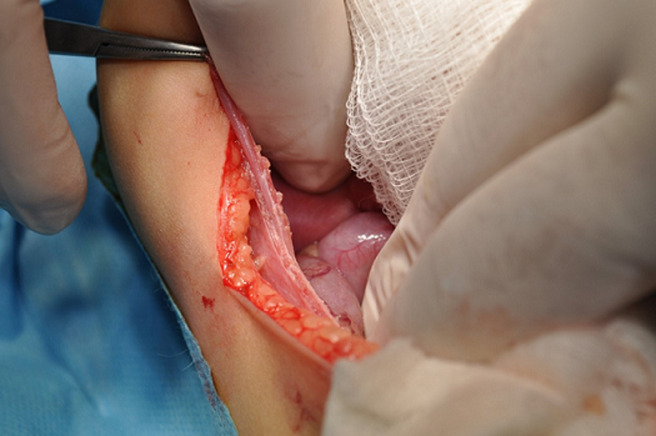
herniated intestinal loops

**Figure 4:**
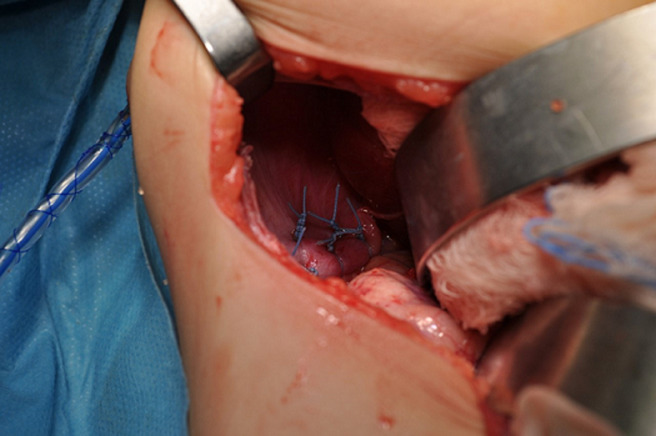
restoration of the diaphragmatic defect according to the Mayo technique; insertion of a chest tube through the sixth intercostal space

With a transverse incision in the right upper quadrant, we accessed the abdominal cavity and found a defect in the right diaphragm (Figure 2, Figure 3). The loops of the whole small bowel and the loops of the colon till the median of the transverse colon were gently retracted from the right hemithorax back to the abdominal cavity through the diaphragmatic defect. The bowel did not present signs of strangulation, the liver dome was misaligned to the medium line and the right coronary ligament of the liver was found hypoplastic. After the retraction of the abdominal viscera in their normal cavity, we aspirated approximately 150ml of serous pleural fluid from the right thoracic cavity and then inserted a chest tube through the sixth intercostal space. We then closed the defect of the diaphragm according to the Mayo technique and reassessed the retracted bowel which seemed to be normal (Figure 4). It must be underlined that a hernia sac was not found during the operation. We finally closed the abdominal trauma in layers and the child was transferred intubated in the pediatric intensive care unit (PICU) in a stable clinical condition. The infant remained at the PICU for five days. The bowel sounds were present 2 days postoperatively and she had a bowel evacuation at the third postoperative day. The patient was extubated at the same day and returned to our Paediatric Surgery Clinic in good condition at the fifth postoperative day. During the hospitalization in our department, the infant remained in good condition. The nasogastric tube was removed the day that she returned from the PICU, the breastfeeding started and was well tolerated apart from two episodes of vomiting the next two days. The chest tube was removed at the sixth day postoperatively and the intravenous antibiotic administration with a second generation cephalosporin was stopped. The next day and because of the association of diaphragmatic hernias with other congenital anomalies, a consultation from a paediatric cardiologist was asked because the other systems were checked through the preoperative CT scan. The heart ultrasound revealed that the anterior descending and the circumflex branch of the left coronary artery appear to protrude from separate orifices in the left atrium of the Valsalva, which is a finding of no particular clinical significance. The child was discharged at the seventh postoperative day in an excellent general condition and was then followed up at outpatient clinic 15 days, 1, 6, 12 and 24 months postoperatively. She had a full recovery from the operation, with no wound infections and without difficulty to thrive. At the time that this case is reported, the child is healthy and has not presented any medical problem associated with her past surgical history.

## Discussion

In Bochdalek hernias, also known as posterolateral diaphragmatic hernias or pleuroperitoneal hernias, the anatomical defect is in the posterolateral part of the diaphragm allowing abdominal viscera to herniate into the thoracic cavity [1,5]. BH results from an improper closure of the foramen of Bochdalek, which is a small opening through the fetal diaphragm connecting pleural and peritoneal cavities, which normally closes between 7 and 10 weeks of gestation [7]. The failure of closure of this foramen causes a persistent diaphragmatic defect, allowing abdominal viscera to be trapped in the thorax [1,6]. The absence of prenatal diagnosis and the belated diagnosis of BH can be explained by the protective role of liver or spleen which may occlude small diaphragmatic defects and prevent the bowel herniation during fetal development [8]. Although most CDHs present in first 24 hours of life, 5-25% of the patients may have a late presentation [3]. The heterogeneity of the symptoms that a patient with a late presented BH presents, such as recurrent pulmonary infections or atypical gastrointestinal symptoms which vary from gastroesophageal reflux disease to severe and life threatening conditions such as gastric or bowel volvulus, small bowel or colon obstruction, incarceration, or strangulation makes the diagnostic procedure challenging for a paediatric surgeon [2,3]. BH are predominantly left sided (~80%), rarely right sided (~20%), and there are descriptions in the literature of bilateral located hernias [5]. An interesting feature of the BH is the high incidence of coexisting associated anomalies such as congenital heart disease, Down´s syndrome, trisomy 18 and malrotation [4]. This, as well as the occurrence of this congenital defect in twins with very similar congenital anomalies raise the possibility that diaphragmatic hernias may result from an inheritable defect [9].

Besides the fact that there is a consensus that BH has to be surgically repaired when is diagnosed even if it remains asymptomatic because of the fatal conditions that may arise and cannot be prevented if it is left untreated, a still controversial point in the management of those patients is the surgical approach, whether transthoracic or transabdominal [5,6]. We report the first case of an undiagnosed right sided BH that our department encountered during the last 15 years with such a severe clinical presentation. The fact that the onset of the symptoms was late, the atypical signs and the coexistence of a head injury led our team to a face a difficulty in the differential diagnosis. The deterioration of the clinical status of our patient during the examination at the emergency department because of cardiorespiratory distress, did not provide us the time to use the plain chest and abdomen x-ray in order to establish the diagnosis. We need to underline that in such emergent situations that the patient history is vital for the diagnosis, it may be disorientating such in our case. The parents´ description for the head injury at the same day, combined with the quick deterioration of the clinical condition, the respiratory distress and the loss of consciousness led us to use the full body CT scan in order to establish the diagnosis besides the fact that the child did not presented obvious skin lesions on her head. The wide spectrum of the differential diagnosis included both brain injury, undiagnosed heart disease and gut volvulus, which means that we could not focus our diagnosis on a system which pathology gave those signs and symptoms. Our patient had a unilateral right sided BH with no other associated anomalies, which was not prenatally diagnosed and presented, in terms of emergency, with acute respiratory distress.

The surgical approach that our team preferred was the transabdominal access because of the length of the bowel that was incarcerated in the right hemithorax as the CT scan revealed, and the high possibility of bowel necrosis preoperatively. The transabdominal approach through an upper transverse incision allows easier reduction of the incarcerated viscera from the thoracic cavity, facilitates possible surgical manipulations such enterectomy and anastomosis performance and gives the surgeon the opportunity for a detailed inspection of the rest abdominal contents and a simultaneous inspection of the contralateral hemidiaphragm for the presence of bilateral hernia. Marhuenda *et al*. describe both transthoracic and abdominal approach as accepted techniques for diaphragmatic hernia repair. They underline that while transthoracic approach offers better exposure of the surgical field, facilitates easier liberation of adhesions, identification of the phrenic nerve and resection of the hernia sac, the transabdominal approach facilitates the reduction of the incarcerated viscera and simultaneously gives the surgeon the opportunity to identify and treat a possible malrotation or bilateral hernia [2]. Besides the fact that our patient investigation did not reveal associated anomalies, we advocate that when a diagnosis of a BH is conducted through a chest and abdominal x-ray and not through CT scan, the surgeons have to complete the investigations with heart and abdominal ultrasound preoperatively or postoperatively according to the patient´s clinical condition. Finally, we propose a close follow-up of the children patients postoperatively twice a year for the first postoperative year and once a year until the adulthood, which includes clinical examination and chest x-ray when the thorax auscultation raises concern. We highly recommend that the patient´s parents must be informed in detail for the possibility of late postoperative complications such postoperative ileus, in order to be vigilant and do not ignore the symptoms that may arise.

## Conclusion

Prenatally undiagnosed congenital diaphragmatic Bochdalek hernia with late onset is an extremely rare entity in the childhood population. The usually misleading symptoms and signs make the diagnosis difficult and tricky. When it presents with acute but non-specific symptomatology, it makes the differential diagnosis both difficult and risky. We advocate the surgical repair through the transabdominal approach when the diagnosis is established and the use of CT scan preoperatively, when the symptoms are atypical and there is a doubt for the diagnosis with the use of a plain x-ray. Among paediatric specialties, a high index of suspicion is recommended to obviate delay in diagnosis which could be fatal. The associated anomalies have to be investigated with a thorough clinical examination and the appropriate radiological techniques.

## References

[1] Hu X, Liu B (2018). Bochdalek hernia. The Lancet. Lancet Publishing Group.

[2] Marhuenda C, Guillén G, Sánchez B, Urbistondo A, Barceló C (2009). Endoscopic repair of late-presenting morgagni and bochdalek hernia in children: case report and review of the literature. J Laparoendosc Adv Surg Tech.

[3] Sanh W, Langer JC, Ratnapalan S (2017). Congenital diaphragmatic hernia in a child with abdominal pain and respiratory distress. Pediatr Emerg Care.

[4] McGivern MR, Best KE, Rankin J, Wellesley D, Greenlees R, Addor MC (2015). Epidemiology of congenital diaphragmatic hernia in Europe: a register-based study. Arch Dis Child Fetal Neonatal Ed.

[5] Bagłaj M (2004). Late-presenting congenital diaphragmatic hernia in children: a clinical spectrum, Pediatric Surgery International. Springer Verlag.

[6] Kirby E, Keijzer R (2020). Congenital diaphragmatic hernia: current management strategies from antenatal diagnosis to long-term follow-up, Pediatric Surgery International. Springer.

[7] Gilbert A, Cardos B (2017). Tension gastrothorax as a complication of bochdalek Hernia. J Emerg Med.

[8] Zefov VN, Almatrooshi MA (2015). Chest X-ray findings in late-onset congenital diaphragmatic hernia, a rare emergency easily misdiagnosed as hydropneumothorax: a case report. J Med Case Rep.

[9] Kazez A, Bakal U, Tartar T, Ersoz F, Colakoglu Y, Gurbaz M (2012). Congenital diaphragmatic hernia in identical twins. J Indian Assoc Pediatr Surg.

